# Comparison of tracheal tube cuff pressure with two technique: fixed volume and minimal leak test techniques

**DOI:** 10.15171/jcvtr.2019.08

**Published:** 2019-03-13

**Authors:** Sarvin Sanaie, Farzad Rahmani, Sara Chokhachian, Ata Mahmoodpoor, Jafar Rahimi Panahi, Robab Mehdizadeh Esfanjani, Masomeh Mirzaei, Hassan Soleimanpour

**Affiliations:** ^1^Aging Research Institute, Tabriz University of Medical Sciences, Tabriz, Iran; ^2^Emergency Medicine Research Team, Tabriz University of Medical Sciences, Tabriz, Iran; ^3^Students’ Research Committee, Tabriz University of Medical Sciences, Tabriz, Iran; ^4^Anesthesiology Research Team, Tabriz University of Medical Sciences, Tabriz, Iran; ^5^Neurosciences Research Center, Tabriz University of Medical Sciences, Tabriz, Iran; ^6^Road Traffic Injury Research Center, Tabriz University of Medical Sciences, Tabriz, Iran

**Keywords:** Endotracheal, Emergency Department, Airway Management

## Abstract

***Introduction:*** There is a correlation between endotracheal cuff pressure and airway complication; therefore, cuff pressure measurement is of an essential importance. The gold standard technique is measuring the cuff pressure by a calibrated manometer. However, there are several methods that injects air into balloon pilot and measures the cuff pressure. The aim of this study is to compare the tracheal cuff pressure measurement by two methods: fixed volume and minimal leak test (MLT).

***Methods:*** This descriptive study was performed at the emergency department on 110 patients. Patients were randomized into two groups. For one group, fixed volume technique and for the other group MLT was used.

***Results:*** Mean cuff pressure was 46.07±23.54 cmH2O in the fixed volume group and 33.72±9.14 cmH2O in the MLT group (*P*=0.05) which is significantly higher in the fixed volume group (*P*=0.028). In addition, 56.4% and 78.2% of the subjects had normal cuff pressure in the fixed volume group and MLT group, respectively; indicating a significantly higher rate in MLT group (*P*=0.025).

***Conclusion:*** Both techniques cause above normal intracuff pressure; however, MLT produces more acceptable pressure than fixed volume. It seems that the volume of 10 cc produces high pressures; therefore, fixed values may yield more appropriate results in lower volumes.

## Introduction

The use of a cuffed endotracheal tube (ETT) is essential for a patient who needs protected airway.^1-3^ ETT cuff pressure management is an important step in the management of airway after endotracheal intubation especially in critically ill patients who undergo mechanical ventilation. Insufficient cuff pressure causes pulmonary aspiration of oropharyngeal content and excessive amounts of cuff pressure leads to decreased tracheal capillary perfusion.^4-7^ There is a correlation between cuff pressure and airway complication in patients.^8^ Cuff is designed to prevent the aspiration and allows for the application of positive pressure ventilation, so adequate cuff function and pressure is crucial for this intervention. An ideal pressure range is defined to be 20-30 cmH_2_O and both under- and over-inflation of the ETT cuff can cause various complications in the patient. There are several methods for air injection into balloon pilot and measurement of cuff pressure. The gold standard technique is measuring the cuff pressure by a calibrated manometer (analog vs digital/ intermittent vs continuous) which is recommended for pediatrics and adults,^9-13^ but is not widely used and a routine practice in Iran. The injection of fixed amount of air by a syringe into endotracheal cuff is the most routine way. This technique is easy, simple and not expensive, but the correlation between cuff to tracheal wall pressure and the volume of air is not linear and occasionally leads to cuff distention and over pressure.^14-16^ In emergency situation, it can be an appropriate method for application of secure airway but it could be dangerous and leads to different complication during time.^17,18^ Harvie and colleagues in a cross-sectional study assessed the association between the cuff manometry and minimal leak test (MLT). They enrolled 45 mechanically ventilated patients for more than 3 months. There was no relationship between cuff pressures and any measured variables. They showed that MLT causes both over- and under-inflation of the ETT cuff, and other techniques like cuff manometry should be employed.^19^ Carhart et al in their study purposed to define the optimal cuff inflation volume to achieve the 20-30 cmH2o cuff pressure. They showed that the cuff inflation volume range of 6-7 mL resulted in the highest likelihood of achieving the desired cuff pressure range, while cuffs inflated with 8-10 mL resulted in dangerously high cuff pressures in all instances. In the absence of a more ideal solution, the results of this study suggest that narrowing the recommended cuff inflation volume from 5-10 mL to 6-7 mL might be a reasonable target for any tube size.^20^ Based on the different methods and various results, we designed a trial to compare the tracheal cuff pressure measurement by two methods: fixed volume and MLT.

## Materials and Methods

This descriptive study was performed at the emergency department of Imam Reza hospital during the year 2017. The inclusion criteria consisted of all patients requiring intubation. Exclusion criteria were patients aging under 18 years and subjects unwilling to give written informed consent. The following information was noted for each patient: gender, age, vital signs, cause of intubation, tracheal tube size, and intra cuff pressure. Random Allocation software was used for randomization of patients in one block model and two groups. For one group Fixed volume technique and for the other group MLT was used. The ETT which we used was high volume, low pressure type (Supa. Company, Iran). The ETT cuff was filled with 10 cc of air in the fixed volume technique, and in the MLT technique, after intubation, patients were positioned supine on a 30° inclined bed. Oropharynx and ETT were suctioned and tube cuff fully inflated. Then air withdrawn slowly from cuff with auscultation over trachea until a leak was heard. Cuff volume recorded as MLT. All cases were checked using the standard pressure control method with manometer. Both techniques were applied by single emergency medicine specialist. Tracheal tube cuff pressures were measured by Mallinckrodt manometer. The cuff pressure was adjusted by manometer immediately after inflating in both techniques. When the cuff pressure was higher than normal. We reduced the cuff pressure after using manometer. The data of both groups were compared with the standard pressure of the tracheal tube cuff (20-30 cm H_2_O). The sample size was calculated based on the results of previous studies. It was 22 for fixed volume technique^11^ and 35 for MLT.^21^ Considering the 95% confidence interval and power of 80, 51 samples in each group were calculated using G power software. To increase the accuracy of the study, the total sample size was elevated to 110. Data were analyzed using SPSS version 16. Independent sample *t* test and Mann-Whitney U test were used to compare the quantitative data and chi square to compare the qualitative data between two groups. *P* value less than 0.05 was considered as the significant level.

## Results

Data from 110 patients (55 subjects in each group) was collected during the study period. Demographics and baseline data are shown in Table 1. The mean age of the patients was 63.28±21.07 in the fixed volume group and 60.62±21.83 in the MLT group; there was no significant difference between two groups (*P* = 0.869). Of all participants, 67 were men and 43 were women, with no significant difference between the groups (*P* = 1.322). Mean body temperature was not significantly different between two groups (*P* = 0.854). The main cause of intubation in the fixed volume group was loss of consciousness (81%) and in the minimum leakage group was poisoning (27.3%); the causes of intubation are shown in Table 2.

**Table 1:** Demographic characteristics and baseline data of patients in two groups

**Variable**	**Fixed volume method**	**MLT**	***P*** ** value**
Age, mean ± SD	63.28±21.07*	60.62±21.83	0.869
Sex (M/F)	36/19	31/24	0.327
BT	37.60±0.24	37.15±0.74	0.854

* MLT: minimal leak Technique; BT: body temperature.

**Table 2:** Causes of intubation in two groups

**Cause of intubation**	**Fixed volume method**		**MLT**	
	**No.**	**%**	**No.**	**%**
Loss of consciousness	39	81.3	5	9.1
Respiratory failure	7	14.6	0	0
Seizure	1	2.1	0	0
Trauma	1	2.1	0	0
Embolism	0	0	2	3.6
Loss of consciousness	0	0	10	18.0
Stroke	0	0	3	5.5
Reduced oxygen saturation	0	0	1	1.8
Tachypnea	0	0	2	3.6
Acidosis	0	0	2	3.6
Toxication	0	0	15	27.3
Sepsis	0	0	4	7.3
COPD	0	0	2	3.6
Pneumonia	0	0	4	7.3
Shock	0	0	1	1.8
ARDS	0	0	1	1.8
SAH	0	0	1	1.8
ICH	0	0	1	1.8
Respiratory distress	0	0	1	1.8

MLT: minimal leak Technique; COPD: chronic obstructive pulmonary disease; ARDS: acute respiratory distress syndrome; SAH: subarachnoid hemorrhage; ICH: intracranial hemorrhage.

Mean cuff pressure was 46.07±23.54 cmH2O in the fixed volume group and 33.72±9.14 cmH2O in the MLT group (*P* = 0.05) which is significantly higher in the fixed volume group (*P* = 0.028). Comparison of cuff pressure between two groups is shown in Table 3. In this regard, 56.4% and 78.2% of the subjects had normal cuff pressure in the fixed volume group and MLT group, respectively; indicating a significantly higher rate in MLT group (*P* = 0.025).

**Table 3:** Comparison of intracuff pressure between two groups

**Group**	**Intracuff pressure**			
	**Normal**		**Abnormal**	
	**No. **	**%**	**No. **	**%**
Fixed volume method	31	56.4	24	43.6
MLT	43	78.2	12	21.8
Total	74	67.3	36	32.7

## Discussion

This study aimed to compare the ETT cuff pressure between two methods of MLT fixed volume technique and showed that the mean cuff pressure in the fixed volume group was significantly higher than the MLT group. Moreover, cuff pressure in more than 40% of cases in fixed volume group was in abnormal ranges. Therefore, it can be concluded that cuff pressure has more suitable values in MLT than the fixed volume method. Galinski et al assessed the incidence of intracuff excessive pressure in the transferred or out-of-hospital patients and revealed that the majority of cuff pressures exceeded safe pressure of 14 to 27 cm H2O.^11^ Al-Metwalli et al performed a study on 75 adult patients scheduled for nitrous oxide-free general anesthesia to compare three common methods of inflating the ETT cuff (precise standard pressure, sealing pressure, or finger estimation). They concluded that cuff pressure was significantly lower in the sealing group and higher in the finger group compared to the control group.^22^ In a study conducted by Khan et al on 100 adult patients, ETT cuff inflation was performed using two syringes sizing either 10 mL or 20 mL. They showed that higher cuff pressure was achieved when the cuff was inflated with 20 mL syringe compared to the 10 cc syringe. Nevertheless, the cuff pressure measurements were above the standard in both groups and monitoring of cuff pressure by manometer was suggested.^23^ In the present study, we concluded that the cuff pressure was in the standard range in approximately 70% of cases which is higher than the values in the study conducted by Saleh Moghadam et al (i.e. 20%).^24^ A study on 40 patients in ICU setting demonstrated that minimal occlusion volume method caused more appropriate cuff pressure than estimating method but VAP (ventilator-associated pneumonia) incidence was not significantly different in the two groups because cuff pressure was more than 20 cmH_2_O in both groups.^25^ Although statistically significant differences were seen in systolic and diastolic blood pressures and respiratory rates between two groups, it does not appear to have clinical significance. A study conducted on patients having cardiac surgery revealed that manual cuff pressure measurement compared to MLT significantly reduced the hoarseness after extubation but did not have a significant effect on the sore throat.^21^ In this study, cuff pressure manometer was used for monitoring the pressure of cuff in one group and it was maintained around 20-30 cm H_2_O during 15 minutes before the cardiopulmonary bypass. In the other group, only MLT was done for inflation and the cuff pressure was measured using the manometer but no intervention was done. It was concluded that measurement of intracuff pressure is necessary for avoidance of over or under inflation of the cuff. Keeping the cuff pressure in 20-30 cmH2O will prevent loss of volume during ventilation and some complications such as hoarseness, sore throat, and tracheomalacia. A study by Liu et al on 509 patients scheduled for elective surgery under general anesthesia from 4 tertiary care university hospitals in Shanghai, China, measured the mean cuff pressure by palpation of the pilot balloon. It was 43±23.3 mm Hg before adjustment and 20±3.1 mm Hg after adjustment (*P*<0.001). Controlling the ETT cuff pressure by a manometer decreased some complications like hoarseness of voice, sore throat, cough and bloody sputum even in short duration procedures (1–3 hours).^26^ Comparison of the accuracy of the estimation of endotracheal cuff pressure by finger palpation with cuff pressure measurement using a device by 20 ICU team members showed that estimation by finger palpation had a low accuracy and precise intracuff pressure measurement by the use of objective devices is mandatory to prevent complications of over- or underinflation.^27^ A study by Braz et al on 85 adult patients showed that about 55% of cuffs in the intensive care unit, 90% of cuffs after anesthesia without nitrogen oxide in post-anesthetic care and 45% of cuffs after anesthesia with nitrogen oxide in post-anesthetic care have a pressure more than 40 cmH_2_O. This study showed that estimation methods cannot measure high pressures and all of the examined cuffs have more than normal pressures, especially in patients in whom nitrous oxide have not been used for anesthesia.^28^

The study of Maleki et al on 50 patients admitted to the intensive care unit indicated that 76% of cuffs had a pressure more than 30 cmH_2_O and mean cuff pressure was 53.40 ± 25.42 cmH_2_O having a significant relationship with patients’ body temperature.^29^

A study by Mousavi and colleagues on 30 patients admitted to the intensive care unit Showed that the most reason for hospital admission was due to brain lesions and the main cause of intubation was respiratory support after brain lesions. In addition, this study revealed that the cuff pressure was outside the normal range in 49% of patients reaching to 18.5% six hours after the correction.^30^ The study of Hoffman et al. In 2005 on 41 academic staffs of emergency medicine showed that experience had a very little impact on the ability to estimate the cuff pressure and the participants were only able to diagnose the cuffs with excessive pressure with a sensitivity of 22%.

This suggests that even academic staffs of emergency department cannot be accurately inflate the cuff or estimate the cuff pressure.^31^ A study by Parwani et al on 53 paramedical staff indicated that the participants were not able to inflate the cuff properly; the average generated pressure was >108 cm H(2)O. On the other hand, they were not able to detect the cuffs with excessive intracuff pressure; only 13% of them could detect overinflated ETT cuffs. This study also showed that 41% of the causes of intubation was related to changes in consciousness, 29% to heart failure, 16% to respiratory distress, 10% to hemodynamic instability and 6% due to other cases^32^; which is similar to the results of present study. The study of Saleh Moghaddam et al on 70 intubated patients in the intensive care unit and emergency department showed that 80% of cases had an abnormal cuff pressure and in 51.6% of cases cuff pressure was in excessive ranges. Causes of intubation was pulmonary disorders in 55.2% of cases. There were also a significant relationship between Cuff pressure and body temperature.^24^ Atlas in 2005 provided a mathematical model for the relationship between body temperature and cuff pressure.^33^ In 2014, Sharifi et al showed that a high percentage of cuffs had excessive intracuff pressures. They also showed that there is no specific relationship between cuff pressure and body temperature.^34^ Rose and Redl in a survey of cuff management practices in ICUs in Australia and New Zealand showed that the preferred method was cuff pressure measurement which was used exclusively or combined other methods.

Cuff pressure measurement was exclusively used in 20 ICUs (22%) and minimal occlusive volume technique was exclusively used in 16 ICUs (17.5%)

Most ICUs used minimal occlusive volume technique after intubation rather than for ongoing management. The minimal leak technique was used only in one ICU (1%) after intubation.^35^ The study of Bolzan et al on 267 patients showed that use of the volume-time curve compared to minimal occlusive volume for ETT cuff management significantly reduced the complications such as sore throat, cough, and chest pain in the coronary artery bypass graft.^36^ Taslimi et al assessed the ETT cuff pressure and time intervals measurements among 61 intensive care units patients. They indicated that the cuff pressure was normal in 16.4% cases at the first step and after 6 hours, cuff pressure adjustment increased by 78.7%. Despite 6 hours cuff pressure control, the range of misregulation was 21.3%. Therefore, cuff pressure should be measured at shorter intervals to prevent complication due to over and under inflation.^37^ The sample size of our study was small and this was an important limitation of study. In conclusion, both techniques cause above normal intracuff pressure, 20-30 cmH_2_O, in the present study; however, MLT produces more acceptable pressure than fixed volume. Considering the results of this study, as well as the studies on fixed volume technique, it seems that the volume of 10 cc produces high pressures.

## Ethical approval

The ethics committee of Tabriz University of Medical Sciences have approved and registered the study under the code number 5/D/37863 on July 24, 2016.

## Competing interests

None.

## Acknowledgments

The authors are grateful to all participated in the study, in addition to data collectors, supervisors, and administrative staff of Emergency Medicine, Emam Reza hospital, Tabriz University of Medical Sciences, Tabriz, Iran. This article is based on a dataset forming part of Sara Chokhachian’s M.D thesis, entitled “Comparison of Tracheal Tube Cuff Pressure with two Technique: Fixed Volume and minimal leak Techniques.” It was registered at Tabriz University of Medical Sciences (No: 95/1-8/1) in 2016.
